# Local-nonlocal assisted multifunctional photonic crystals

**DOI:** 10.1038/s41377-026-02308-3

**Published:** 2026-05-19

**Authors:** Wenjing Lv, Haoye Qin, Xiaodong Shi, Fulong Shi, Xinyang Mu, Zhou Zhou, Jiazheng Qin, Bo Li, Cheng-Wei Qiu, Qinghua Song

**Affiliations:** 1 Tsinghua Shenzhen International Graduate School, Tsinghua University, Shenzhen, China; 2 Department of Electrical and Computer Engineering, National University of Singapore, Singapore, Singapore; 3 A*STAR Quantum Innovation Centre (Q.InC), Agency for Science, Technology and Research (A*STAR), Singapore, Singapore

**Keywords:** Metamaterials, Photonic crystals

## Abstract

Metasurfaces excel at local, spatially varying control of wavefronts, whereas photonic crystals (PhCs) are admired for their nonlocal resonances such as bound states in the continuum (BICs). These two regimes—local control and nonlocal collective response—have long been viewed as difficult to integrate within a single platform. Here, we introduce local-nonlocal assisted multifunctional PhCs unifying wavefront shaping and BICs by embedding meta-notches within PhC pillars. The locally tunable notches generate spectral-zero-assisted topological phase for efficient 2π coverage, while the strongly confined BIC modes remain largely unperturbed, preserving high-*Q* nonlocal resonances. This constructive local-nonlocal integration synthesizes the design freedom of metasurfaces with the dispersive resonance of PhCs in a single planar device. Our approach extends the capabilities of flat optics, enabling multifunctional PhCs and opening pathways toward higher-order topologies, advanced imaging, communication, and analogue optical computing.

## Introduction

Photonic crystals (PhCs) in optics have emerged as one of the most outstanding platforms for studying resonances^1,2^, band structures^3,4^, polarization control^5–7^, and topological physics^8–13^. Conventionally, PhCs require perfect periodic structures to provide highly prominent and clear resonances outstanding from non-resonant features. Among the intriguing phenomena in PhCs is the bound state in the continuum (BIC) enabled through symmetry engineering or parameter tuning^14–16^, which manifests as a special nonradiative resonance with vanishing spectral linewidth^17,18^ and topological nature^19–22^, showing strong dependence on transverse momentum.

Despite advantages, the spatial invariance of nonlocal PhCs imposes constraints on design flexibility and device functionality. Their momentum- and angle-dependent responses contrast sharply with metasurfaces, which exploit spatially varying unit cells to achieve local, position-dependent wavefront control^23–26^. This practical separation arising from the momentum-dependent responses of PhCs and the spatially local variation of metasurfaces has historically hindered the realization of a unified platform. As a result, the accessible parameter space in planar photonics remains severely limited, precluding the engineering of exotic optical responses such as higher-dimensional fields^27^, higher-order topologies, and multifunctionality within a single, robust device^28,29^.

Here, we resolve this conflict by demonstrating multifunctional PhCs that robustly unify local and nonlocal effects through embedding meta-notches within PhC pillars. The locally tunable notches induce spectral-zero-assisted topological phase encirclement for efficient 2π coverage, achieved with minimal perturbation, while the confined BIC modes remain mostly unaffected, preserving high-*Q* nonlocal resonances. This approach synergistically combines the design freedom of metasurfaces, which provide singular topological control for arbitrary wavefront manipulation, with the resonance stability of PhCs, which maintain largely unperturbed nonlocal BICs. The result is a unified planar platform that simultaneously supports both functionalities, as confirmed experimentally by far-field meta-holograms and measured dispersion band structures.

This work unlocks previously inaccessible local-nonlocal degrees of freedom in metasurfaces and PhCs, enabling deterministic control of both wavefront shaping and collective resonance. It establishes a framework for multifunctional flat optics, in which singular and BIC topologies are co-integrated within a single device. Such PhCs provide a versatile platform for realizing higher-order topologies and dual-purpose meta-systems in advanced imaging, communication and display technologies.

## Results

**Fig. 1:**
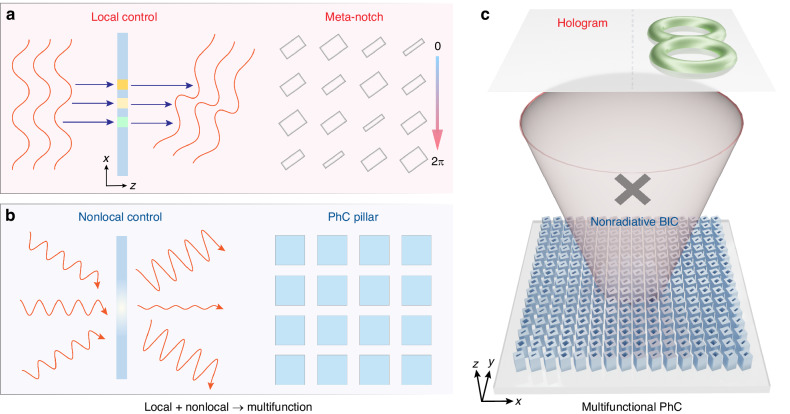
Concept of local-nonlocal PhC unifying wavefront shaping and BIC. **a** A metasurface leverages local control through spatially varied meta-atoms to achieve position-dependent wavefront control. **b** A PhC supports nonlocal control through responses from extended spatial regions, enabling angle-dependent behavior and dispersive resonance. The multifunctional PhC integrates these local and nonlocal functionalities within a single planar structure: meta-notches with variable widths generate full 2π local phase modulation via topological encirclement around a spectral singularity, while the PhC pillars preserve nonlocal BIC resonances. **c** By combining both functionalities, the multifunctional PhC thus simultaneously supports nonradiative BIC with vanishing linewidth and singularity-assisted topological phase for arbitrary meta-hologram generation

In Fig. 1a, a metasurface exploits local degrees of freedom by using spatially varied meta-atoms for position-dependent wavefront control. By contrast, a PhC harnesses nonlocal degrees of freedom, with responses arising from an extended spatial region that enable angle-dependent behavior and high-*Q* resonances (Fig. 1b). These two regimes represent distinct optical control modalities—local control and nonlocal collective response—that have largely remained separate. In our approach, they are ultimately unified: meta-notches with tunable widths provide full 2π local phase modulation assisted by topological encirclement around a spectral singularity, while the remaining high-index pillars simultaneously sustain nonlocal BIC resonances.

We therefore embed local metasurface functionality into a periodic PhC lattice, preserving the invariant high-index pillars while varying the inner low-index notches. As shown in Fig. 1c, this architecture not only sustains nonradiative BICs with strong angular dependence but also creates singular topology that enables meta-holograms with arbitrarily encoded phase profiles. It is noteworthy that the topological properties impart robustness against fabrication imperfections and structural deviations, thereby preserving the performance of the multifunctional PhC and relaxing fabrication constraints. The resulting meta-notch embedded PhC embodies an elegant local-nonlocal integration, merging complementary optical functionalities into a single platform for enhanced free-space control and advanced topological photonic devices.

**Fig. 2:**
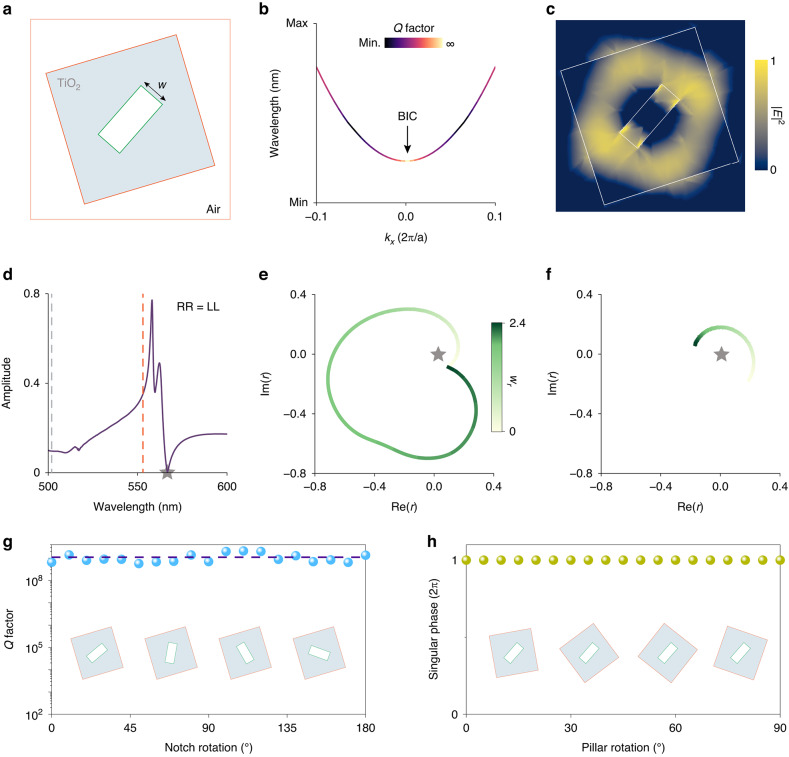
Simulated results of robust local and nonlocal control. **a** The unit cell, shown in cross-section at the half-height of the PhC pillar, consists of a nanopillar made of TiO_2_ and a notch with a width of *w*. **b** Band structure of one eigenmode shows the existence of BIC with infinite *Q* factor. **c** Field distribution of the nonlocal BIC eigenmode, showing strongly confined profile and implying resilience against inner notch perturbation at the central field minimum. **d** Simulated reflection spectrum under circularly polarized light, revealing a zero-amplitude singular point near 560 nm (indicated by a star). **e** Near 550 nm (red line in **d**), increasing notch width *w* *=* *w*_*r*_*w*_*0*_ leads to the encirclement around the spectral zero with 2π phase, where *w*_*0*_ = 55 nm. The horizontal and vertical axes represent the real and imaginary parts of the complex reflection coefficient, respectively. **f** Away from the singularity (grey line in **d**), there is no topological encirclement and no full 2π phase coverage. **g**, **h** Robustness of nonlocal and local properties. Thanks to preserved inversion symmetry, *Q* factor maintains infinite regardless of notch rotations (**g**). The singularity induced topological phase can always cover 2π as topological encirclement for arbitrary pillar rotation (**h**)

Figure 2a illustrates the unit cell with a period of 360 nm, consisting of a 600-nm-thick TiO_2_ nanopillar with a side length of 250 nm, incorporating an embedded notch. The width *w* of the notch can be controlled for the phase modulation. For the PhC eigenmode, the calculated band structure reveals the emergence of a BIC resonance (Fig. 2b). The corresponding field profile exhibits strong confinement and indicates robustness against perturbations introduced by the central notch, owing to a field minimum at the notch location (Fig. 2c). At a notch width of 70 nm, the simulated reflection spectrum reveals a spectral zero under circular polarization near 560 nm in Fig. 2d, corresponding to a topological defect characterized by nontrivial phase winding^16,30,31^. By gradually increasing the notch width, we demonstrate that at a wavelength slightly detuned from this spectral singularity, the system undergoes a full 2π phase accumulation (Fig. 2e), effectively encircling the singularity and inducing a topological evolution. To clarify the physical origin of this effect, the 2π phase evolution arises from the topological winding of the complex reflection coefficient around a spectral-zero singularity. The meta-notch provides the tuning parameter that perturbs the scattering channels. As the notch width varies, the reflection amplitude encircles the spectral zero in the complex plane, enabling a full 2π phase accumulation. This mechanism represents a singularity-assisted topological phase distinct from conventional propagation or geometric phases. The rectangular meta-notch geometry is chosen for its fabrication robustness and its ability to provide a smoothly tunable perturbation parameter while minimally disturbing the BIC mode, which exhibits a field minimum at the notch location. Varying the notch width directly modulates the scattering channels, enabling controlled encirclement of the spectral-zero singularity.

In contrast, for a larger detuning near 500 nm, such encirclement is no longer feasible, resulting in trivial topology (Fig. 2f). The presence of the topological defect enables enhanced phase modulation, allowing the phase to accumulate from 0 to 2π with minimal local notch variations. The topological encirclement is achievable within a finite spectral window in which the singularity exerts dominant influence. As the operating wavelength moves further away from this region, the phase winding weakens, and the system transitions naturally to a non-topological evolution due to insufficient coupling to the singularity.

We also confirm the robustness of both nonlocal and local functionalities in the embedded PhC. The preserved inversion symmetry of the notched pillar ensures that the *Q* factor remains infinite regardless of notch rotation (Fig. 2g), while the singularity-induced topological phase consistently spans 2π via topological encirclement behavior that is largely robust against pillar rotation (Fig. 2h). These capabilities facilitate highly robust local-nonlocal wave control within the multifunctional topological PhC even in the presence of imperfections and deviations. Therefore, this device remains immune to structural rotations and deviations in both the inner notch and outer pillar, highlighting its advantage over delicate designs that require extensive optimization and repeated fabrication. Building on these simulated results, we design meta-holograms by leveraging the notch-assisted singular topology. A modified Gerchberg-Saxton algorithm is developed to convert arbitrary images into target phase distributions, which are then implemented by mapping the local phase profile to the notch width, based on the established relation between topological phase and geometry.

**Fig. 3:**
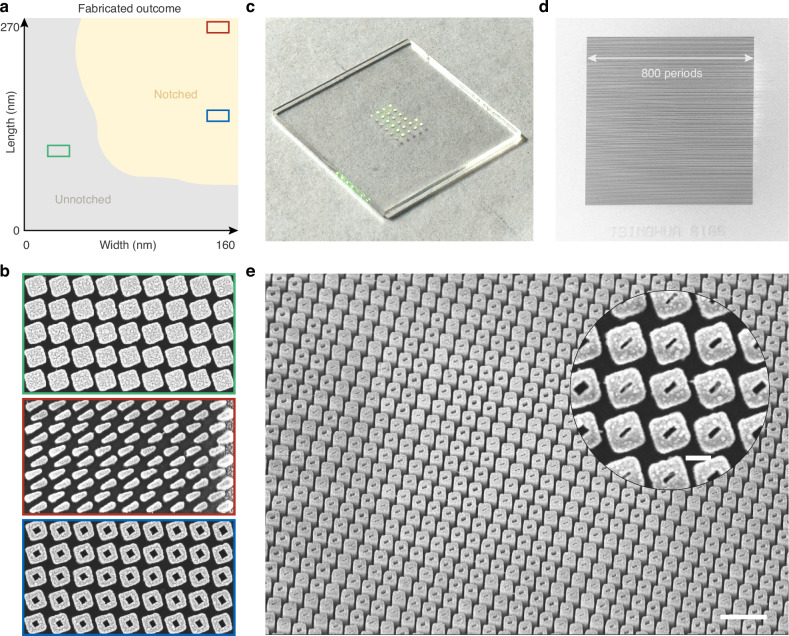
Single-pass fabrication of multifunctional PhCs. **a** Designed meta-notch geometries and fabricated outcome showing notched and unnotched nanopillars due to resolution limit. **b** Three typical outcomes of highlighted region are shown in (**a**) with unnotched, over-notched, and properly notched cases. **c** Photograph of a wafer with multiple embedded PhC samples. **d** Scanning electron microscope image of a fabricated sample having 800 × 800 unit cells. **e** Zoomed-in view of the desired embedded PhC with spatially varying notches. Scale bar, 1 μm

Then we fabricate the sample through standard e-beam lithography process^32,33^. Since the local-nonlocal PhC is a planar device without the requirement of advanced 3D fabrication, the notch and PhC are etched at the same time. We first test a sample with varying designed notch width and length and check the fabricated outcome. To appropriately embed the notch, the designed notch geometries need to fall within a specific region, as shown in Fig. 3a. In the lower-left grey region, the designed notch geometry cannot be embedded due to resolution limit, resulting in pure pillars. Increasing the designed notch length and width enables successful notch fabrication. We show the detailed view of three typical cases of fabricated outcome with unnotched PhC, over-notched PhC, truncating the nanopillar, and properly embedded square notch in PhC (Fig. 3b).

The embedded PhC samples are fabricated on a glass wafer (Fig. 3c) with each size of 800 × 800 unit cells (Fig. 3d). In Fig. 3e, we systematically examine the distribution and structural integrity of the embedded notches within the PhC. By optimizing the notch geometries and overall design parameters, we achieve precise embedding with high uniformity across both the notches and nanopillars. This fabrication strategy enables robust realization of multifunctional PhCs by fully leveraging the notch-induced topological features while maintaining compatibility with standard lithographic resolution and fabrication protocols. Pillar rotation is intentionally introduced as a robustness test. This design choice verifies that the observed 2π phase accumulation is dictated by topological encirclement of the spectral-zero singularity rather than by specific geometric orientations. Moreover, rotation demonstrates compatibility with conventional metasurface design degrees of freedom, while emphasizing that such rotations are not required for phase modulation in this mechanism.

**Fig. 4:**
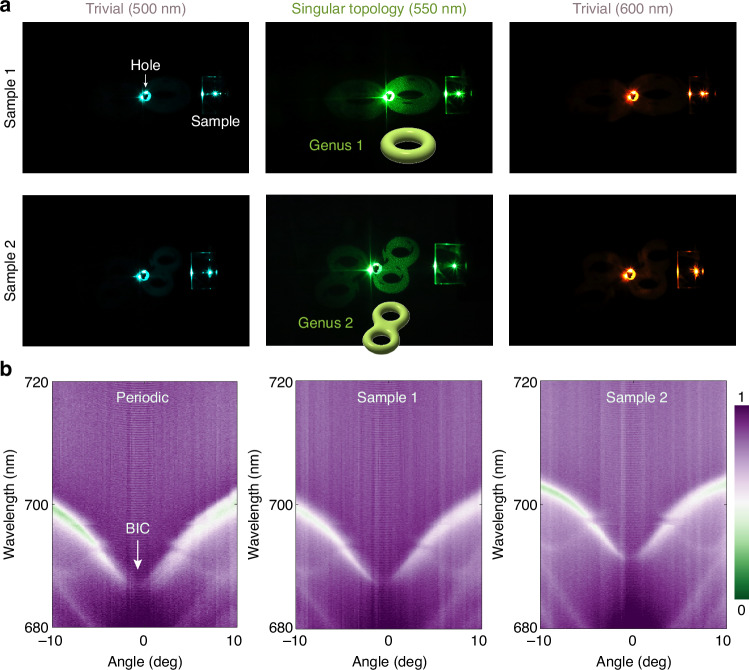
Experimental characterization of local and nonlocal control in multifunctional PhCs. **a** Measurement of meta-holograms of two samples encoded with different genus images. Only near 550 nm, singular topology enables clear demonstration of images, while the spectral detuning eliminates the topological phase. **b** Measured band structure for the existence of nonradiative state at Γ point and angle-dependent response in periodic sample with the same notch and samples 1 and 2 with spatially modulated notch

In experiments, we demonstrated singular-topology-enabled meta-holograms using two samples, each encoding a different image of genus 1 and genus 2 through variations in notch width (Fig. 4a). The sample 2 has a smaller fixed notch length than sample 1 to demonstrate the robustness of singularity-induced 2π phase in the face of deviations, which still robustly persists when increasing the notch width. To demonstrate local wavefront shaping capability from meta-notches, we illuminate the samples with circularly polarized light and capture the reflected beam on a white screen. The reconstructed pattern appears identical for both right and left circular polarizations thanks to the reflection singularity being the same for the two co-polarization channels.

The expected genus images appear around the wavelength of 550 nm with high contrast, confirming the notch-assisted singular topology. As demonstrated previously, the singular topology is highly dispersive, and wavelength detuning deteriorates the topological 2π phase, causing meta-hologram failure at 500 nm and 600 nm. This dispersion also enables topology switching between on and off states through wavelength tuning. It is noteworthy that this singular topology enabled meta-hologram on co-polarized light is in stark contrast to Pancharatnam-Berry phase through geometric rotation of the notch, which occurs on the cross-polarized component^34^ and leads to wavelength independence, low-efficiency, and unavoidable inverted images for opposite circularly polarized incidence.

Moreover, the notch variations present minimal perturbation to the nonlocal PhC’s BIC resonance, which can be verified by experimentally measuring the band structure that provides information across different wavevectors or incident angles. Figure 4b shows the results with fully periodic embedded PhC with the same notch in each nanopillar, and spatially varying notch for the two samples used in Fig. 4a. The vanishing linewidth in the left panel confirms the existence of perfect BIC at the Γ point and reveals strong angle-dependent response for different wavenumbers, the feature rooted in nonlocality. It is worth noting that, although meta-notch variations break exact periodicity, sample 1’s band structure still exhibits the linewidth vanishing phenomenon, whereas sample 2’s band shifts to longer wavelengths due to the reduced notch length while preserving the linewidth disappearance. Therefore, we experimentally verify the nearly unperturbed nonlocal resonant BIC in the presence of notch-assisted singular topology based on the meta-notch embedded PhC, for constructive local-nonlocal integration in a fabrication-friendly planar device. Importantly, relaxing the constraint of strict periodicity does not appreciably compromise nonlocal performance, but instead opens pathways for encoding higher-dimensional information and effects within a single device.

Overall, we propose a new phase modulation mechanism enabled by topological singularity encirclement, in which a full 2π phase coverage is achieved by varying only a single structural parameter. The phase modulation arises from the topological winding of the complex scattering coefficient around a spectral-zero singularity, rather than from geometric rotation or propagation-based mechanisms, making it robust against geometric perturbations and intrinsically dispersive. Moreover, the platform can simultaneously host two independent topologically protected effects: a local, singularity-induced phase topology in parameter space and a nonlocal BIC topology in momentum space.

## Discussion

The demonstration of the local-nonlocal PhC, robustly unifying local singular topology with nonlocal BIC resonances, establishes a practical pathway toward multifunctional devices, higher-order topological effects, and new degrees of freedom in flat optics. This approach enhances wave control through the synergistic integration of local and nonlocal effects, while maintaining fabrication simplicity. For example, local-nonlocal integration could enable complex field structures, including higher-order polarization states and vortex beams. Furthermore, the ability to independently shape nonlocal resonances and local wavefronts suggests a pathway toward analogue optical computing, in which the BIC-supported dispersive response functions as a wave-based operator while the notch-engineered local phases encode the computational input. These directions represent promising next steps for leveraging local-nonlocal integration in multifunctional photonic platforms. Additional local mechanisms, such as general geometric and propagation phases, can be incorporated into the notch, and nonlocal physics driven by diverse band topologies^35,36^ and momentum control^24,37^ can be exploited to realize practical higher-order phenomena and analogue optical computing.

## Methods

### Sample fabrication

The fabrication of the all-dielectric embedded PhC follows a standard electron-beam lithography (EBL) and reactive ion etching (RIE) workflow tailored for TiO_2_. A 600-nm-thick TiO_2_ layer is first deposited onto a glass substrate via electron-beam evaporation to ensure high refractive index and low surface roughness. A PMMA resist is then spin-coated and patterned using EBL to define both the nanopillar footprint and the embedded notch geometry in a single exposure. After development, a chromium hard mask is deposited by electron-beam evaporation and lifted off, yielding a high-fidelity Cr pattern. The TiO_2_ layer is subsequently etched using inductively coupled plasma RIE with optimized CHF_3_/O_2_ chemistry to achieve vertical sidewalls and simultaneous definition of the pillar and notch features. Finally, the Cr mask is removed via wet chemical etching, leaving the fully patterned all-dielectric embedded PhC. This single-pass etching process ensures precise alignment between the notch and the outer pillar while maintaining high uniformity across the 800 × 800-unit-cell arrays.

### Optical setups

The detailed optical setup is shown in Fig. S7. A wavelength-tunable laser (YSL Photonics, China) operating in the visible range is passed through a linear polarizer and a quarter-wave plate to generate circularly polarized light. The laser beam is then weakly focused onto the sample using a lens (LBTEK, China). The reflected holographic images in the far field are projected onto a white screen with a central aperture to allow the incident beam to pass through. The band structure is measured using an angle-resolved spectrometer (IdeaOptics, China) with spectral and angular resolutions of 1.5 nm and 0.5°, respectively.

## Supplementary information

Supplementary

## Data Availability

The data that support the findings of this study are available from the corresponding authors upon reasonable request.
