# Supplementary material for: Local-nonlocal assisted multifunctional photonic crystals
Source: Light Sci Appl. 2026 May 19;15:243. doi: 10.1038/s41377-026-02308-3 (PMC13187152; doi:10.1038/s41377-026-02308-3)
Supplement: Supplementary file 1 — Supplementary [file 41377_2026_2308_MOESM1_ESM.docx]

Supplementary Information for

Local-nonlocal assisted multifunctional photonic crystals

Wenjing Lv^1#^, Haoye Qin^2#^, Xiaodong Shi^3^, Fulong Shi^2^, Xinyang Mu^1^, Zhou Zhou^2^, Jiazheng Qin^2^, Bo Li^1^, Cheng-Wei Qiu*^2*^* and Qinghua Song^1*^

*^1^Tsinghua Shenzhen International Graduate School, Tsinghua University, 518055 Shenzhen, China*

*^2^Department of Electrical and Computer Engineering, National University of Singapore, Singapore 117583, Singapore*

*^3^A*STAR Quantum Innovation Centre (Q.InC), Agency for Science, Technology and Research (A*STAR), Singapore, 138634, Singapore*

*^#^These authors contributed equally to this work: Wenjing Lv, Haoye Qin*

*^*^Corresponding Authors:* [*chengwei.qiu@nus.edu.sg*](mailto:eleqc@nus.edu.sg)*;* [*song.qinghua@sz.tsinghua.edu.cn*](mailto:song.qinghua@sz.tsinghua.edu.cn)

**
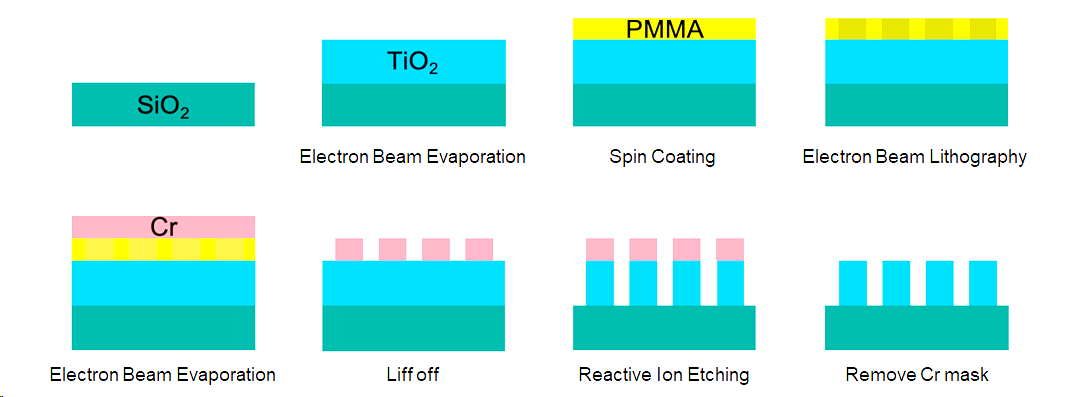
**

**Fig. S1. Fabrication process of the local-nonlocal PhC based on TiO_2_ on glass substrate.** The nanopillars and embedded notches are fabricated simultaneously in a single etching step using standard nanofabrication procedures.

**
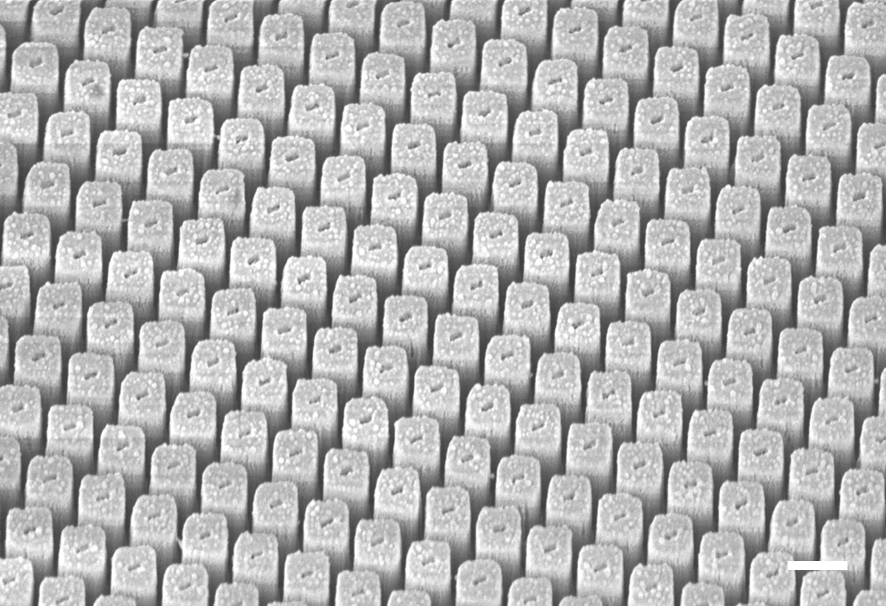
**

**Fig. S2. Scanning electron microscope image of periodic sample with invariant notch geometry.** Regardless of its rotation, the rectangular notch preserves inversion symmetry and supports a perfect BIC. Scale bar, 400 nm.

**
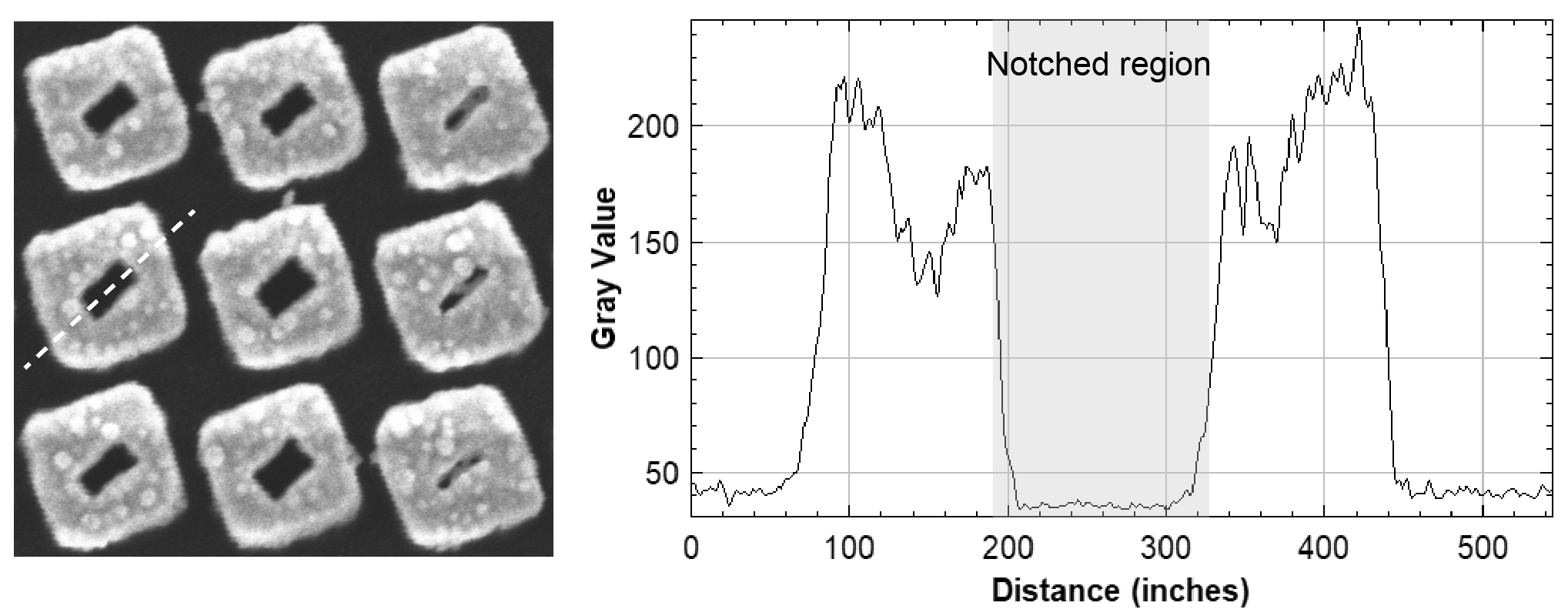
**

**Fig. S3. Scanning electron microscope characterization of notched nanopillar array.** Left: Top-view images of 9 fabricated pillars with embedded notches. Right: Line profile showing image gray values across the notched region.

**
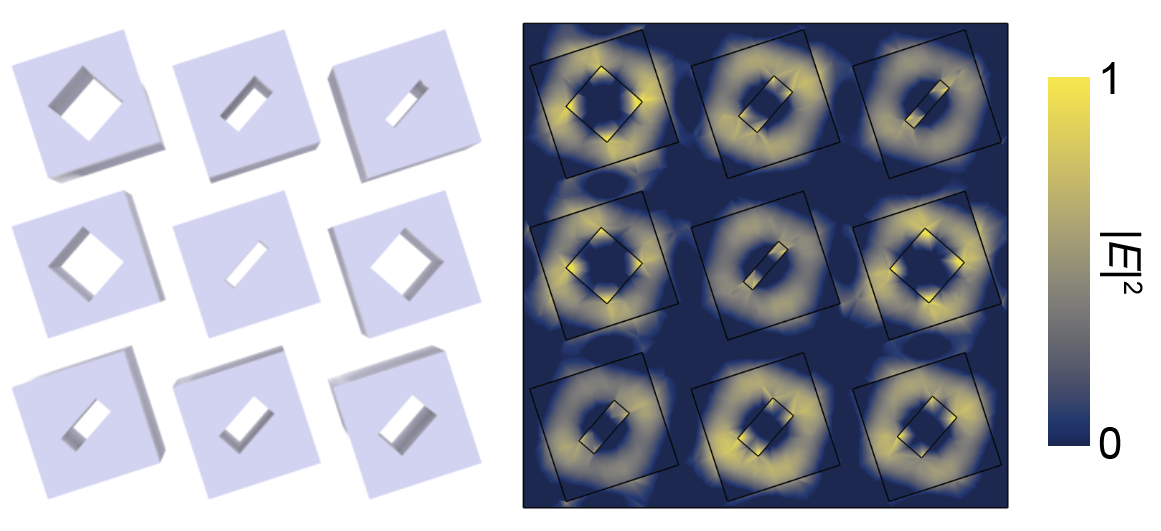
**

**Fig. S4. Nonlocal BIC mode profile in presence of spatially varying notches embedded in nanopillars.** The notch variations minimally disturb the ring-like nonlocal mode since they are embedded within the center, where field intensity is very small.

**
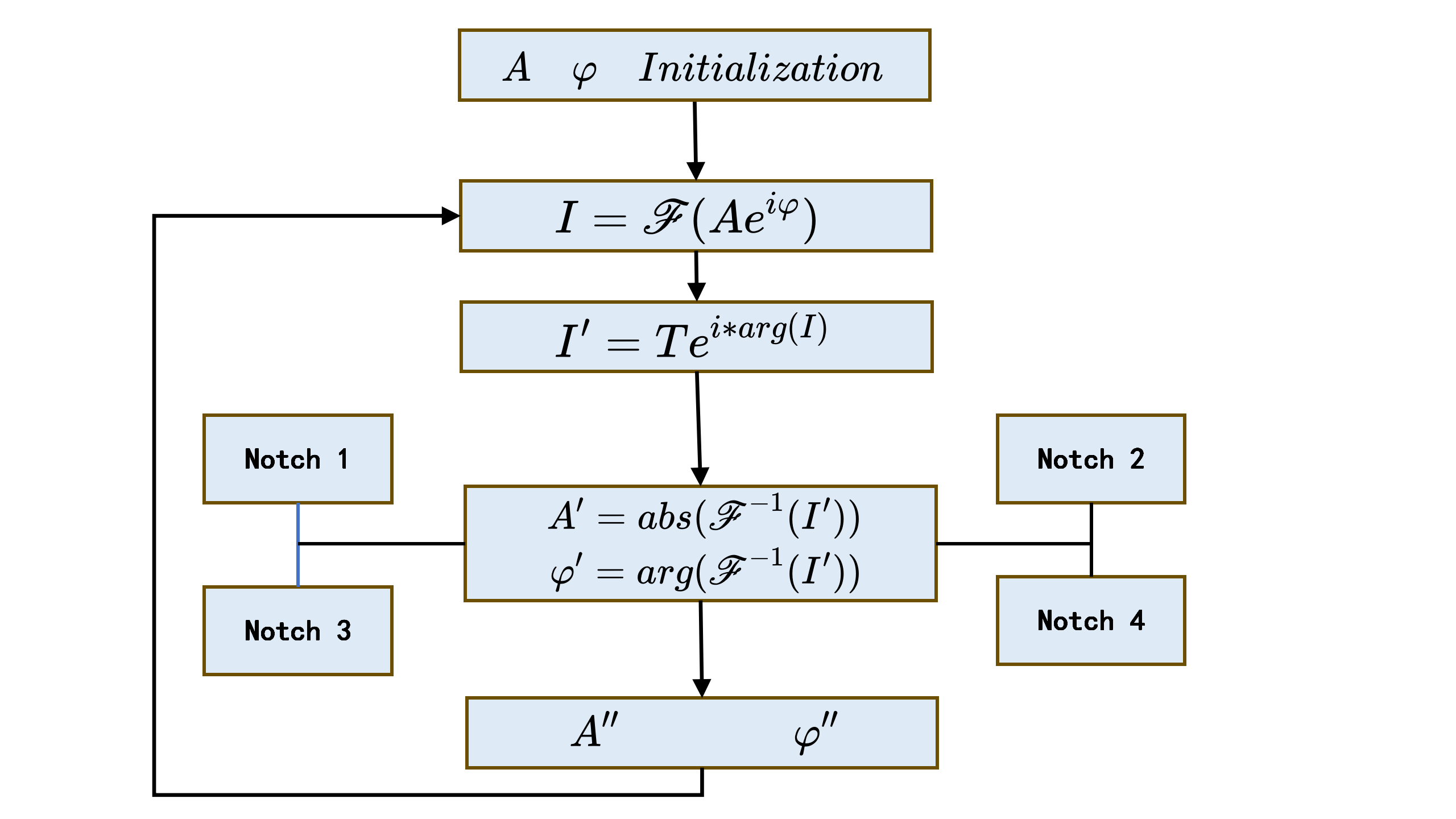
**

**Fig. S5. Flowchart for the meta-hologram encoding algorithm.** The algorithm begins with random initial amplitude and phase distributions (*A* and *φ*) as input. Far-field holograms (*I*) are calculated by applying Fourier transform. The amplitude is then replaced with the target image (*T*), while the phase and amplitude are updated via inverse Fourier transform. The updated phase and amplitude (*A′* and *φ′*) are mapped to four discrete phase elements by minimizing Euclidean distance. The matched phase and amplitude (*A″* and *φ″*) undergo iterative optimization until convergence. Finally, the converged spatial phase distribution yields four notch geometries for implementation.

**
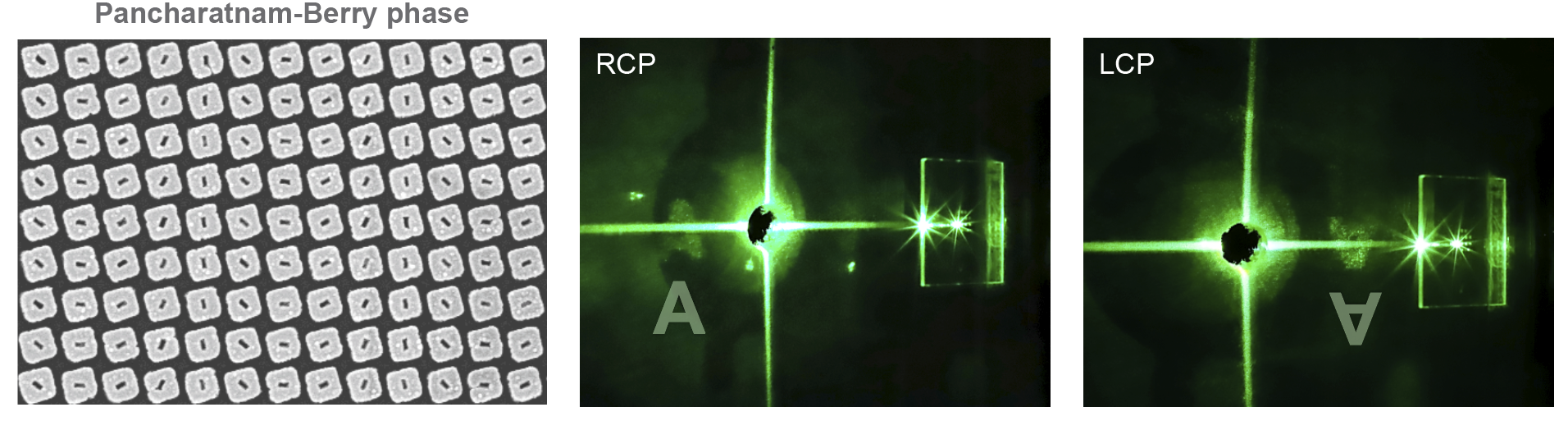
**

**Fig. S6. Rotated notches as Pancharatnam-Berry phase in embedded PhC.** Since the PB phase manifests under cross-polarized light with opposite phase, inverted meta-holograms are generated for right-handed circularly polarized (RCP) and left-handed circularly polarized (LCP) incident light.

**
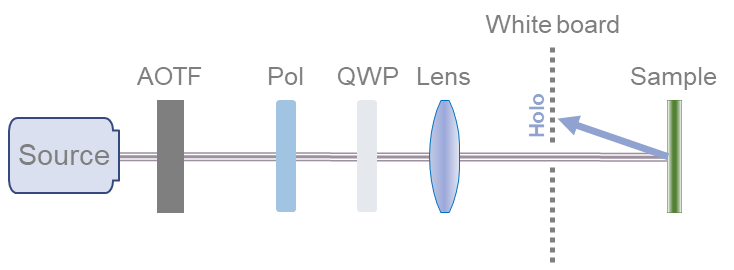
**

**Fig. S7. Optical setup for measuring meta-hologram.** AOTF, acousto-optic tunable filter; Pol, polarizer; QWP, quarter waveplate; Holo, meta-hologram image.


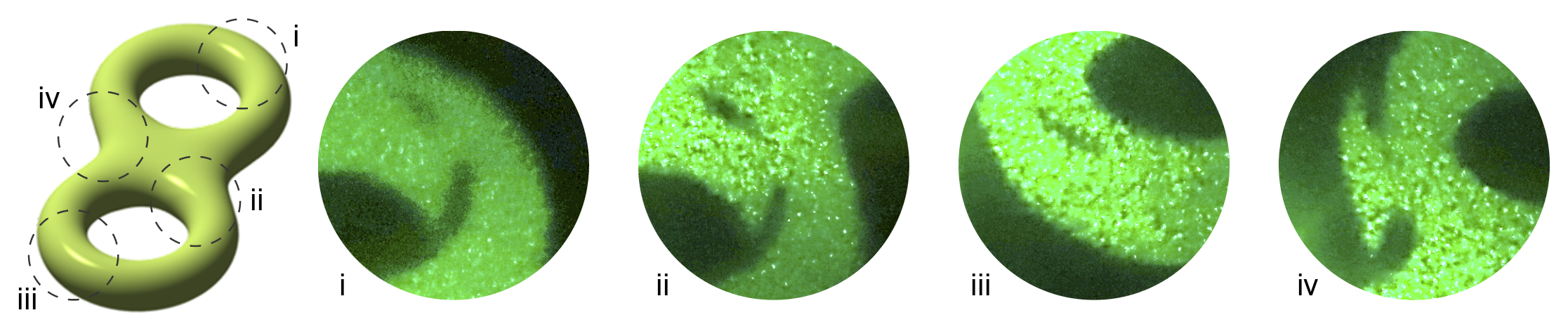


**Fig. S8. Detailed views of meta-hologram via singular topology assisted notch.** The retrieved holograms preserve fine details, as highlighted in the four marked regions.


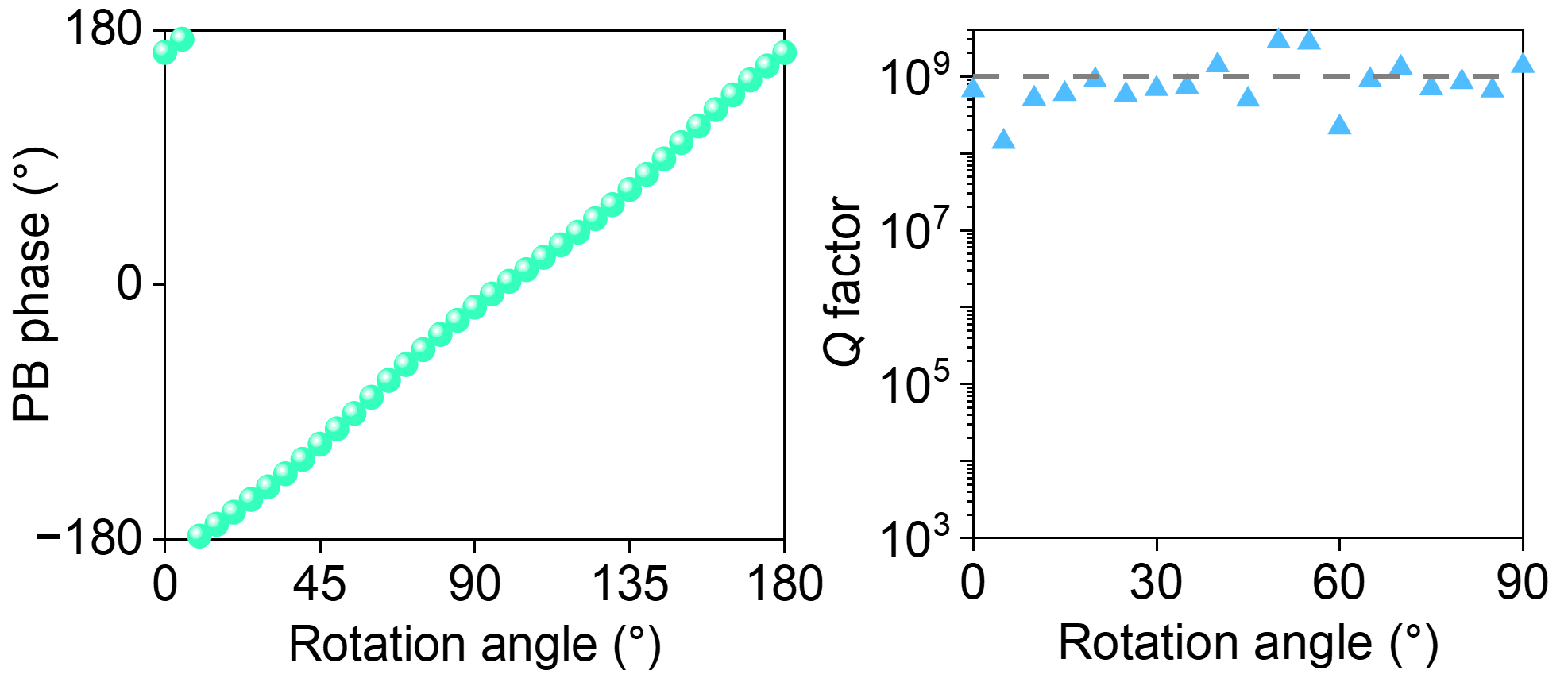


**Fig. S9. Rotation of notch for PB phase and impact of quality factor.** The notch rotation induces anisotropy that enables PB phase coverage of 2π, while the preserved inversion symmetry maintains the quality factor.


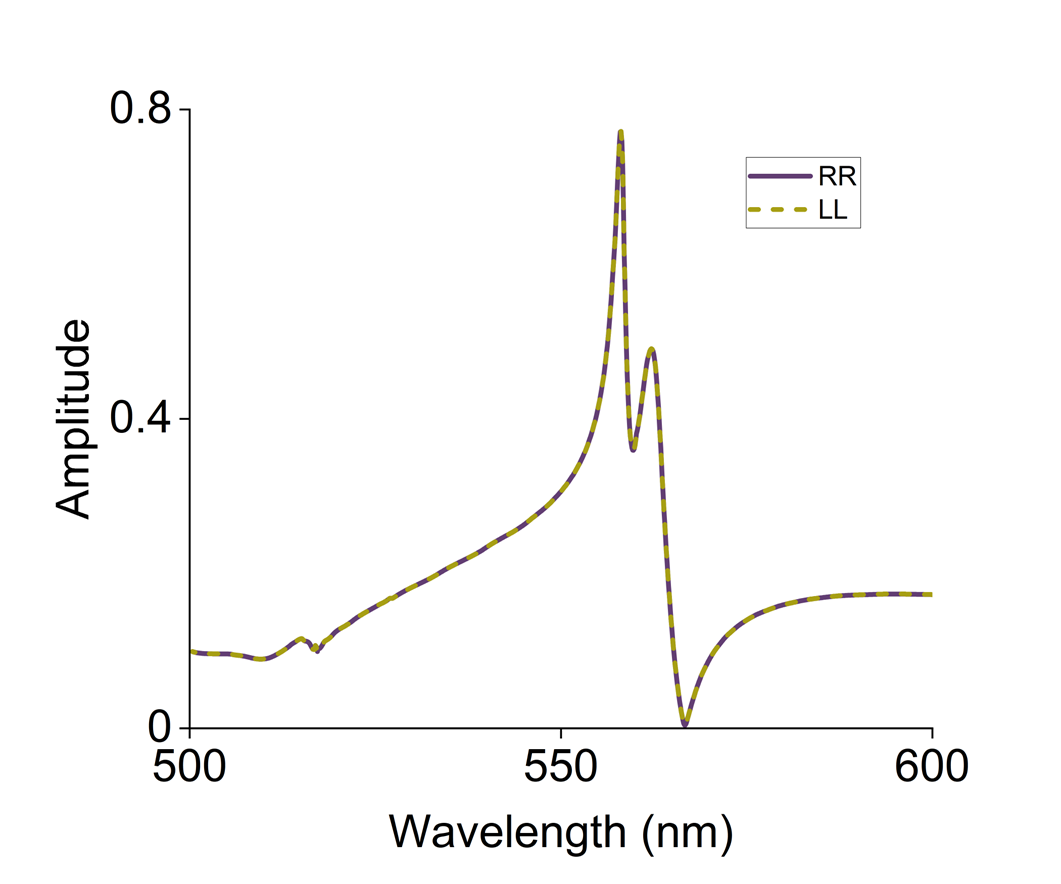


**Fig. S10. Reflection amplitude of co-polarization channels RR and LL.**


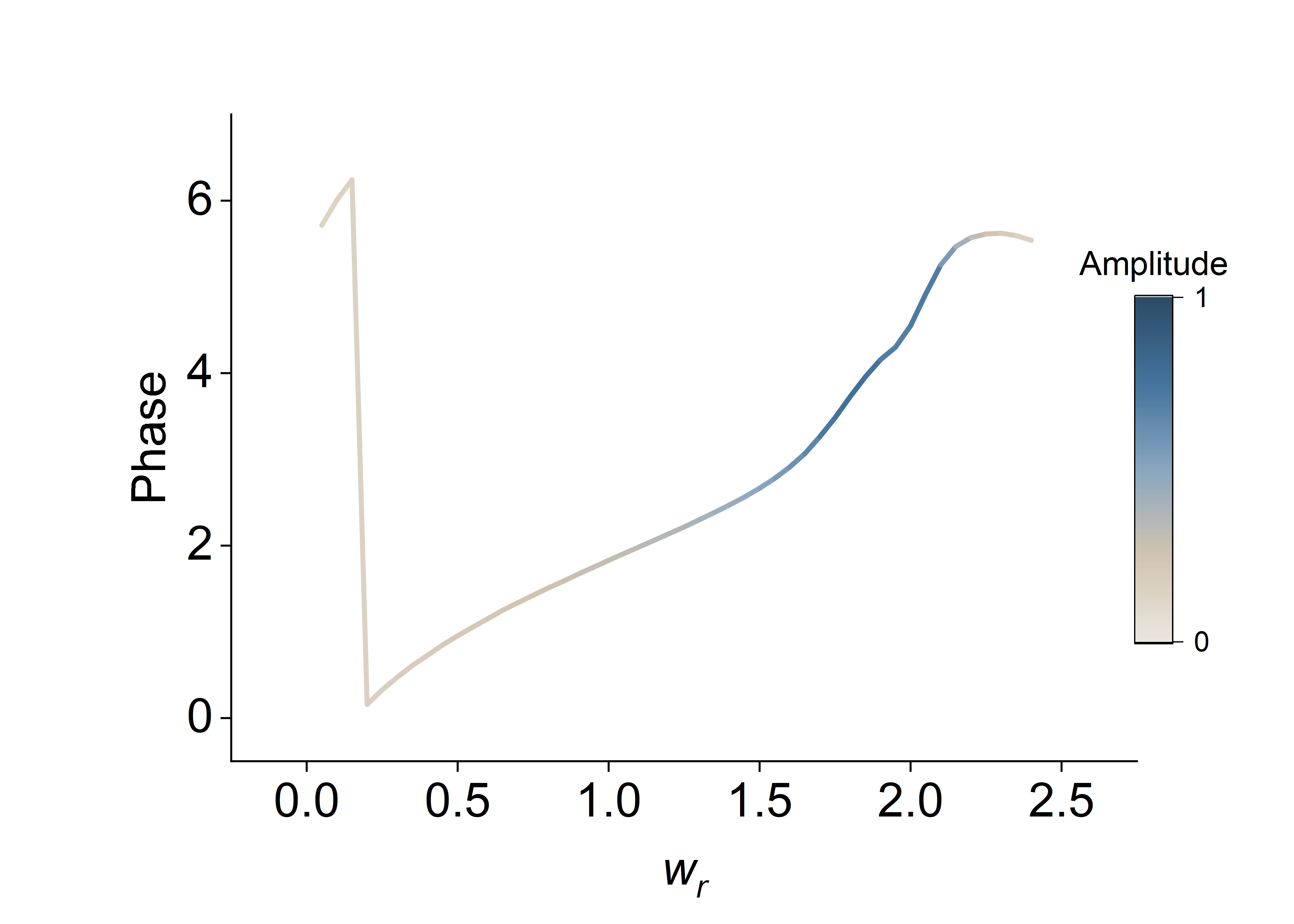


**Fig. S11. Evolution of the reflection phase and amplitude as a function of notch width, showing a full 2π phase coverage enabled by encirclement of the spectral singularity.**


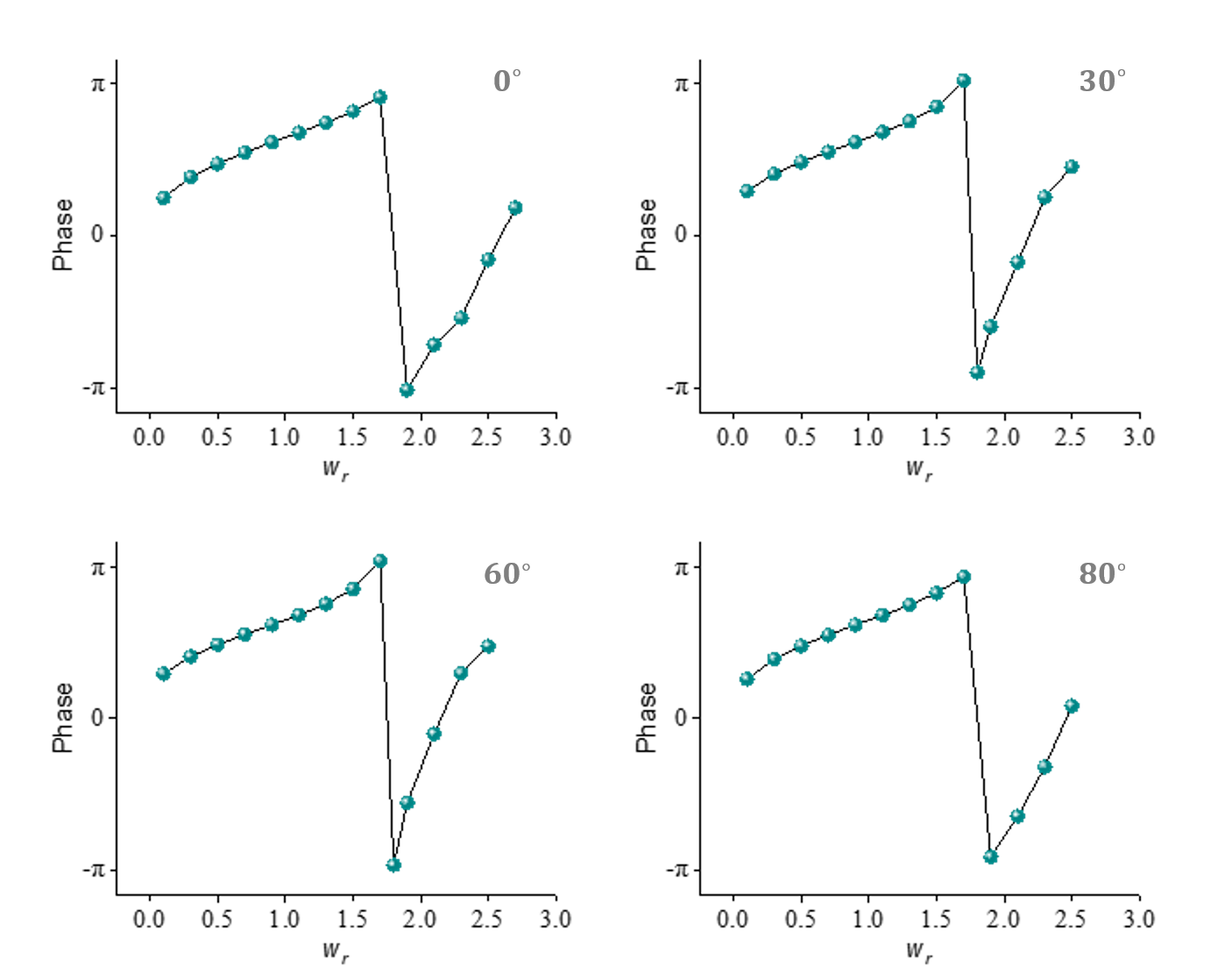


**Fig. S12. Verification of robust topological encirclement around singularity for different pillar rotations.**
